# Supplementary material for: Lipid Cubic Mesophases Combined with Superparamagnetic Iron Oxide Nanoparticles: A Hybrid Multifunctional Platform with Tunable Magnetic Properties for Nanomedical Applications
Source: Int J Mol Sci. 2021 Aug 27;22(17):9268. doi: 10.3390/ijms22179268 (PMC8430948; doi:10.3390/ijms22179268)
Supplement: Supplementary file 1 [file ijms-22-09268-s001.zip › ijms-1348126-supplementary.pdf]

Supporting Information for

Lipid cubic mesophases combined with Superparamagnetic Iron Oxide Nanoparticles: a hybrid multifunctional platform with tunable magnetic properties for nanomedical applications

Lucrezia Caselli [1,2], Marco Mendozza [1,2], Beatrice Muzzi [1,3,4,5], Alessandra Toti [6], Costanza Montis [1,2], Tommaso Mello [7]**;** Lorenzo Di Cesare Mannelli [6], Carla Ghelardini [6], Claudio Sangregorio [1,4,5] and Debora Berti [1,2]*

[1]*Department of Chemistry, University of Florence, Via della Lastruccia 3, Sesto Fiorentino, Florence 50019, Italy;*
[2]*Consorzio Sistemi a Grande Interfase, Department of Chemistry, University of Florence, Sesto Fiorentino, Italy*
[3]*Department of Biotechnology, Chemistry and Pharmacy, University of Siena 1240, I-53100 Siena, Italy*
[4]*ICCOM – CNR, I-50019 Sesto Fiorentino (FI), Italy*
[5]*INSTM, I-50019 Sesto Fiorentino (FI), Italy*
[6]*Department of Neuroscience, Psychology, Drug Research and Child Health – Neurofarba – Section of Pharmacology and Toxicology, University of Florence, Florence, Italy*
[7]*Department of Clinical and Experimental Biomedical Sciences "Mario Serio", Gastroenterology Unit - University of Florence, Florence, Italy*

[*]*Correspondence: debora.berti@unifi.it*

	**Index**	***Page***
**S.1**	*Estimate of the crystallite size from the XRD pattern*	S1
**S.2**	*Magnetization Vs Temperature SPIONs*	S4
**S.3**	*SAXS data elaboration*	S4
**S.4**	*SAXS profiles of bulk mesophases*	S6
**S.5**	*SAXS experiments on cubosomes and magnetocubosomes*	S8
**S.6**	*Generator of Low Frequency Alternate Magnetic Field (LF-AMF)*	S9
S.7	*In Situ structural investigation*	S10
S.8	*Additional images*	S11
S.9	*Bibliography*	S12

S.1 Estimate of the crystallite size from the XRD pattern

Sherrer equation

The Sherrer equation is a formula to determine the size of micro or nano-crystallites from the half-peak width (FWHM) of the X-Ray diffraction profile[1]:

$$\mathrm{d} = \frac{\mathrm{K\lambda}}{\mathrm{Bcos\theta}} \tag{1}$$

where d is the mean size of the crystallite (ordered domains); K, the Scherrer constant, depends on the shape of the crystal, the size distribution and on how the width is determined; it has a value close to unity (here it was taken equal to 0,94); λ is the X-ray wavelength, B is the half-peak width and θ is the Bragg angle.

Néel – Brown relaxation model

The energy barrier of a single domain particle with uniaxial anisotropy is KV, and it becomes increasingly smaller with the decreasing of the volume. Eventually, for particle of few nanometers, the term KV becomes sufficiently small, that, even in the absence of an external field, the thermal energy (k_BT, where k_B is the Boltzman's constant) is sufficient to induce magnetic fluctuations and spontaneous reverse of the magnetization from one easy direction to the other. In these conditions the system behaves like a paramagnet, but with a much higher value of magnetic moment, being the sum of 10^2-10^5 spins; this state is called superparamagnetic state. The temperature at which the system reaches the superparamagnetic state depends on particles volume and anisotropy. We can introduce a relaxation time (τ) for the magnetization reversal process:

$$\tau = \tau_0 \exp\left(\frac{\mathrm{KV}}{\mathrm{k_B T}}\right) \tag{2}$$

where τ_0 is a time constant characteristic of the material and usually is of the order of 10^{-9}-10^{-12} s for non-interacting particles. The magnetic behaviour of single domain particles is then strongly time dependent, i.e. the observed magnetic state of the system depends on the characteristic measuring time of the used experimental technique, τ_m. Therefore, it can be defined a temperature, called blocking temperature (T_B), at which the relaxation time equals the measuring one ($\tau = \tau_m$):

$$T_B = \frac{KV}{k_B \ln\left(\frac{\tau_m}{\tau_0}\right)} \tag{3}$$

Consequently, being T_B dependent on the time scale of the measurements, for experimental techniques with $\tau_m > \tau$ the system reaches the thermodynamic equilibrium in the experimental time window and a superparamagnetic behaviour is observed. Conversely, when $\tau_m < \tau$ quasi-static properties (similar to bulk materials) are obtained, i.e. the particles are in the blocked regime. Thus, a nanoparticles' assembly at a given temperature can be either in the superparamagnetic or blocked regimes, depending on the measuring technique. In particular, for typical ZFC-FC measurement, Equation 3 becomes:

$$T_B = \frac{KV}{25k_B} \tag{4}$$

Using this formula the average magnetic diameter of the nanoparticles was estimated 4.3 nm. It is important to remind that the equations 3 and 4 are obtained for monodisperse and non-interacting single domain nanoparticles, assuming $t_0 = 10^{-9}$. In a system with inter-particles interaction T_B can be increased by the extra energy terms introduced by the dipolar and/or exchange interaction.

S.2 Magnetization Vs Temperature SPIONs

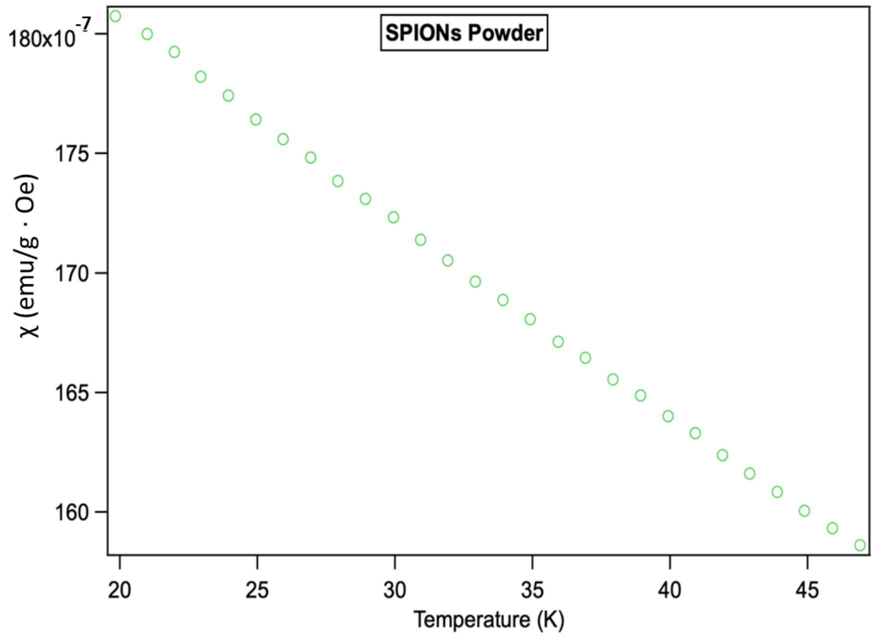

Figure S1 Magnetic susceptibility measurement performed as function of the temperature on SPIONs powder purified by oleic acid and oleylamine capping agents. The intensity of the magnetic field was 10 Oe.

Well above the blocking temperature, the temperature dependence of the susceptibility (and thus of the magnetization) of the SPIONs powder is expected to follow a Curie-Weiss law. In the small temperature range investigated (20-47 °C) such dependence looks like a linear decay. No deviation from this trend is detectable into the range 32-40 °C.

S.3 SAXS data elaboration

The SAXS profile of the SPIONs dispersion in hexane (Figure 1a) was fitted according to the Sphere-Schulz Model by NIST[2,3]. This model calculates the scattering for a polydisperse population of spheres with uniform Scattering Length Density (SLD). The distribution of radii is a Schulz distribution described by the following equation**:**

$$f(R) = (z+1)^{z+1} x^{z} \frac{\exp[-(z+1)x]}{R_{avg}\Gamma(\mathrm{z}+1)} \qquad (S1)$$

where R_{avg} is the mean radius, $x=R/R_{avg}$, z is related to the polydispersity ($p=\sigma/R_{avg}$) through $z=1/p^2-1$. σ^2 is the variance of distribution. The scattering intensity is modeled as follows:

$$I(q) = \left(\frac{4\pi}{3}\right)^2 N_0 \Delta\rho^2 \int_0^\infty f(R) R^6 F^2(qR) dR \qquad (S2)$$

where N_0 is the total number of particles per unit volume, and $\Delta\rho$ is the difference in scattering length density, F(qR) the scattering amplitude for a sphere reported, described by the following equation:

$$F(x) = \frac{3[\sin(x) - x\cos(x)]}{x^3} \qquad (S3)$$

No interparticle interference effects are included in this calculation.

The lattice parameter (d) of cubic and hexagonal phases was calculated as follows:

$$q = \left(\frac{2\pi}{d}\right)\sqrt{h^2 + k^2 + l^2} \qquad (S4)$$

where (hkl) are Miller indices typical of the considered liquid crystalline arrangement.

S.4 SAXS profiles of bulk mesophases

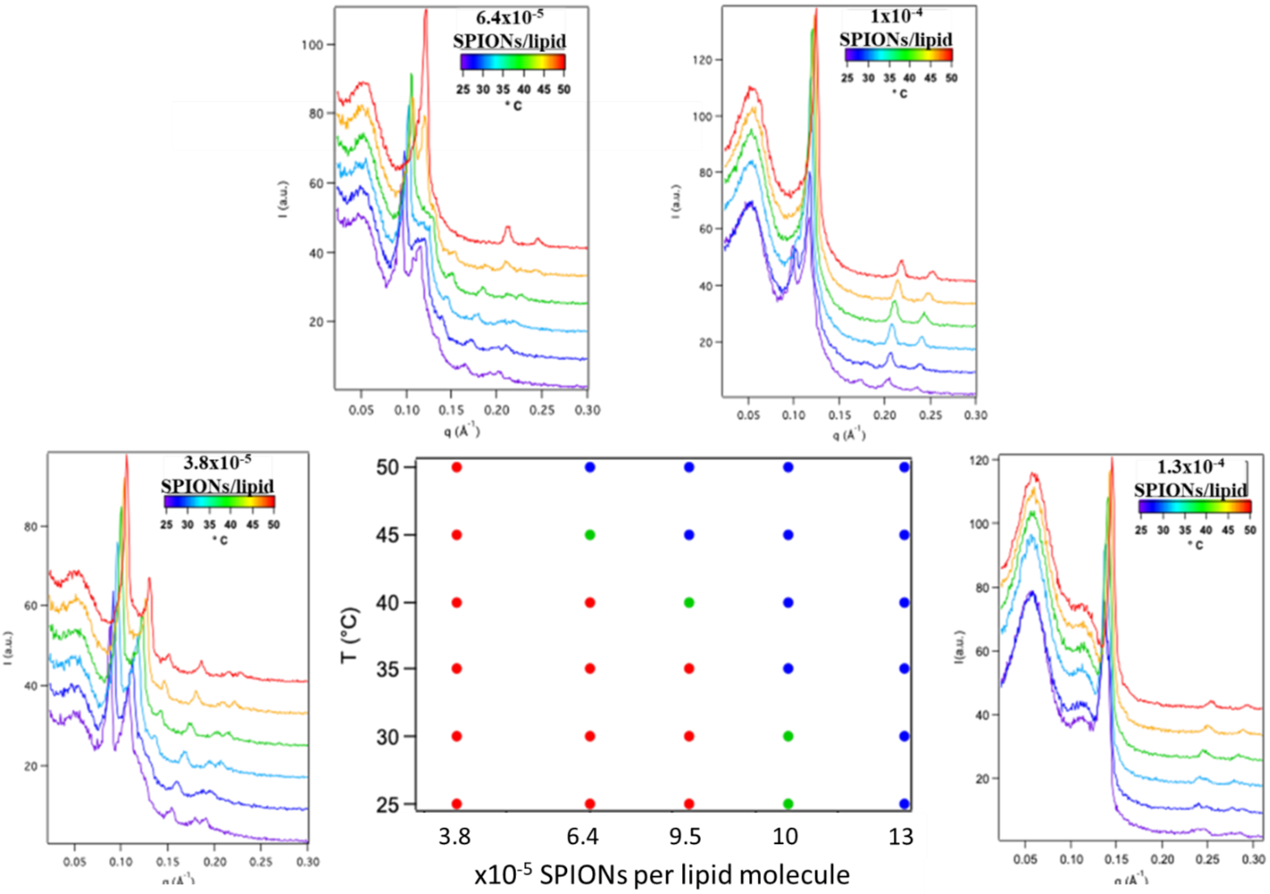

Figure S2 SAXS profiles of cubic phases doped with increasing amount of SPIONs, from $3.8x10^{-5}$, $6.4x10^{-5}$, $1x10^{-4}$, $1.3x10^{-4}$ SPIONs per GMO molecule. The SAXS profile of GMO assembled with $9.5x10^{-5}$ SPIONs per lipid is reported in the main text.
In the middle a phase diagram is reported, showing the type of GMO/SPIONs/water assembly as a function of temperature and SPIONs concentration: red points are associated to Pn3m phase, green points to Pn3m/H_{II} phase coexistence and blu points to the H_{II} phase.

Table S1 Lattice Parameters (Å) of cubic phases doped with different number of nanoparticles

Temperature (°C)	$3.8x10^{-5}$ SPIONs/lipid	$6.4x10^{-5}$ SPIONs/lipid	$9.5x10^{-5}$ SPIONs/lipid	$1x10^{-4}$ SPIONs/lipid	$1.3x10^{-4}$ SPIONs/lipid
25	100 (Pn3m)	94 (Pn3m)	90 (Pn3m)	89 (Pn3m) 54 (H_{II})	46 (H_{II})
30	97 (Pn3m)	91 (Pn3m)	88 (Pn3m)	87 (Pn3m) 53 (H_{II})	46 (H_{II})
35	92 (Pn3m)	87 (Pn3m)	85 (Pn3m)	53 (H_{II})	45 (H_{II})
40	89 (Pn3m)	84 (Pn3m)	83 (Pn3m) 52 (H_{II})	52 (H_{II})	45 (H_{II})
45	86 (Pn3m)	83 (Pn3m) 52 (H_{II})	52 (H_{II})	51 (H_{II})	44 (H_{II})
50	83 (Pn3m)	51 H_{II}	51 (H_{II})	50 (H_{II})	43 (H_{II})

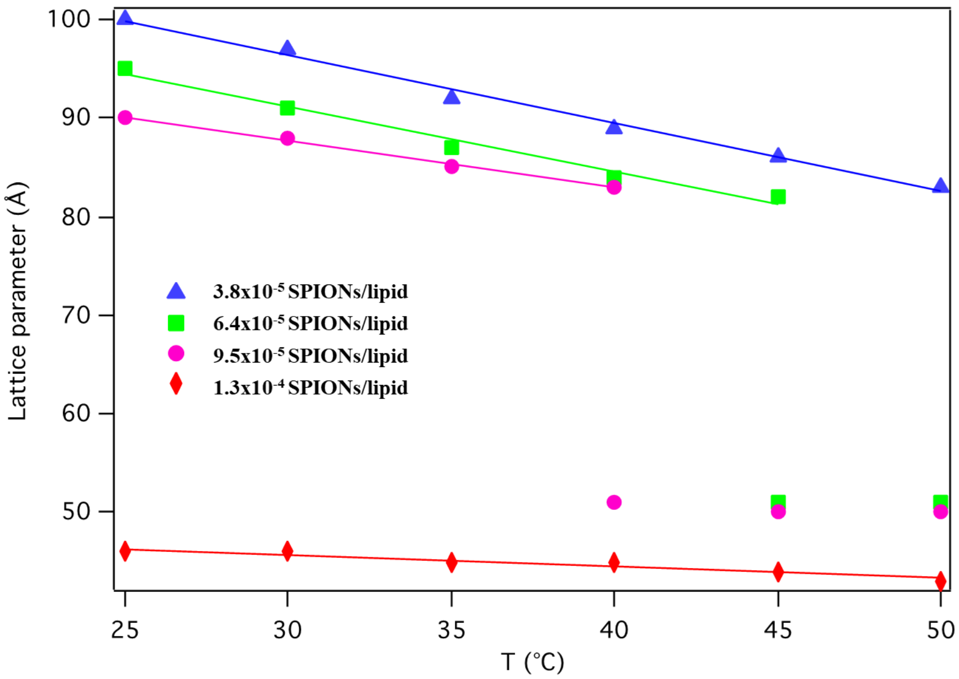

Figure S3 Lattice parameter as function of the temperature for cubic phases doped with $3.8x10^{-5}$, $6.4x10^{-5}$, $9.5x10^{-5}$, $1.3x10^{-4}$ SPIONs per GMO molecule. The slope of linear fit performed into the range of temperature where the structure is preserved, are showed in Table S2.

Table S2 Slope of linear fit showed in Figure S3

Samples	Slope (Å °C^{-1})
**0.12 mM**	-0.69
**0.2 mM**	-0.66
**0.26 mM**	-0.48
**0.39 mM**	-0.12

S.5 SAXS experiments on cubosomes and magnetocubosomes

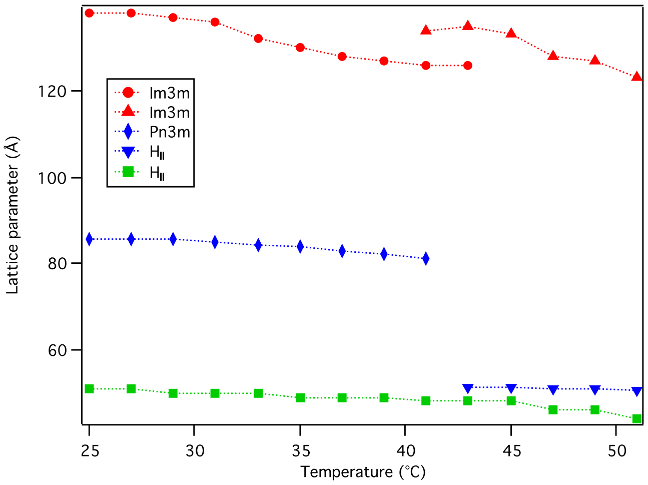

Figure S4 Lattice parameters as function of temperature in the range 25-51 °C for cubosomes (red markers), magnetocubosomes (blue markers) and magnetohexosomes (green markers).

Table S3 Lattice parameter (Å) of cubosomes, magnetocubosomes, magnetohexosomes into the range 25-51 °C

Temperature (°C)	Cubosomes	MagnetoCubosomes	MagnetoHexosome
**25**	138	86 (Pn3m)	51 (H_{II})
**27**	138	86 (Pn3m)	51 (H_{II})
**29**	137	86 (Pn3m)	50 (H_{II})
**31**	136	85 (Pn3m)	50 (H_{II})
**33**	133	84 (Pn3m)	50 (H_{II})
**35**	131	84 (Pn3m)	49 (H_{II})
**37**	128	83 (Pn3m)	49 (H_{II})
**39**	127	82 (Pn3m)	49 (H_{II})
**41**	126 (Im3m Type I)<br>134 (Im3m Type II)	81 (Pn3m)	48 (H_{II})
**43**	126 (Im3m Type I)<br>135 (Im3m Type II)	51 (H_{II})	48 (H_{II})
**45**	133 (Im3m Type II)	51 (H_{II})	48 (H_{II})
**47**	128	51 (H_{II})	46 (H_{II})
**49**	127	51 (H_{II})	46 (H_{II})
**51**	123	51 (H_{II})	44 (H_{II})

S.6 Generator of Low Frequency Alternate Magnetic Field (LF-AMF):

A sinusoidal magnetic field was generated in the gap of a broken toroidal ferrite magnet carrying a solenoid through which an alternating electric current (AC) from a tone generator was led (Figure S5). The samples were placed in the middle of the gap with a Teflon sample holder. Due to the design of the experimental apparatus, the magnetic field inside the cell is not homogeneous and the sample undergoes magnetic field gradients that cannot be avoided. In fact magnetic field amplitudes at different positions were measured by means of a GM-07 Gaussmeter (HIRST Magnetic Instruments Ltd, UK), and found to range from 79.6 to 262.7 kA/m, having a value of 223 kA/m in the middle of the gap of the magnet, i.e. where samples were placed. During the experiments, the field frequency was set at 4.22 kHz.

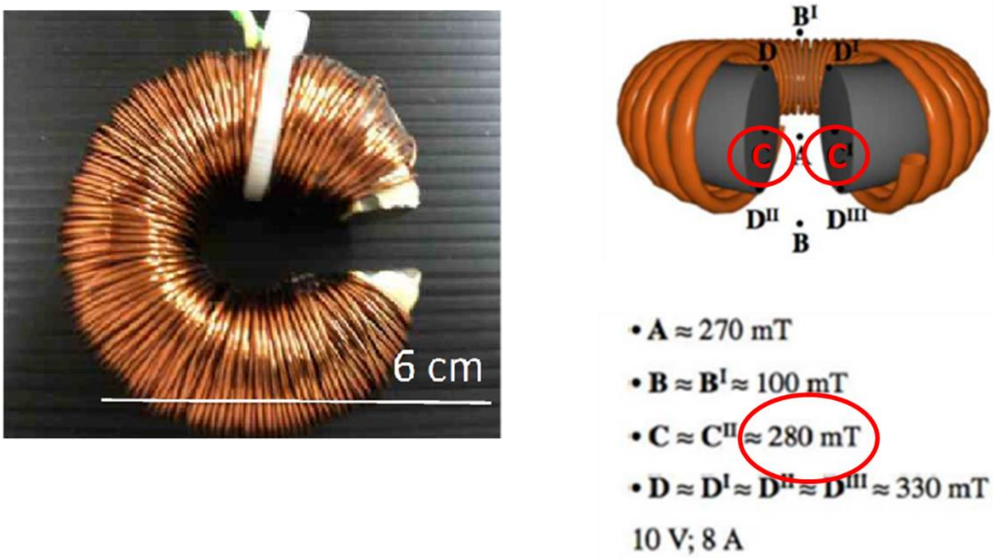

Figure S5 Picture of the broken toroidal magnet used to apply the LF-AMF. Magnetic field values were measured by means of a GM-07 Gaussmeter (HIRST Magnetic Instruments Ltd, UK)

S.7 In Situ structural investigation

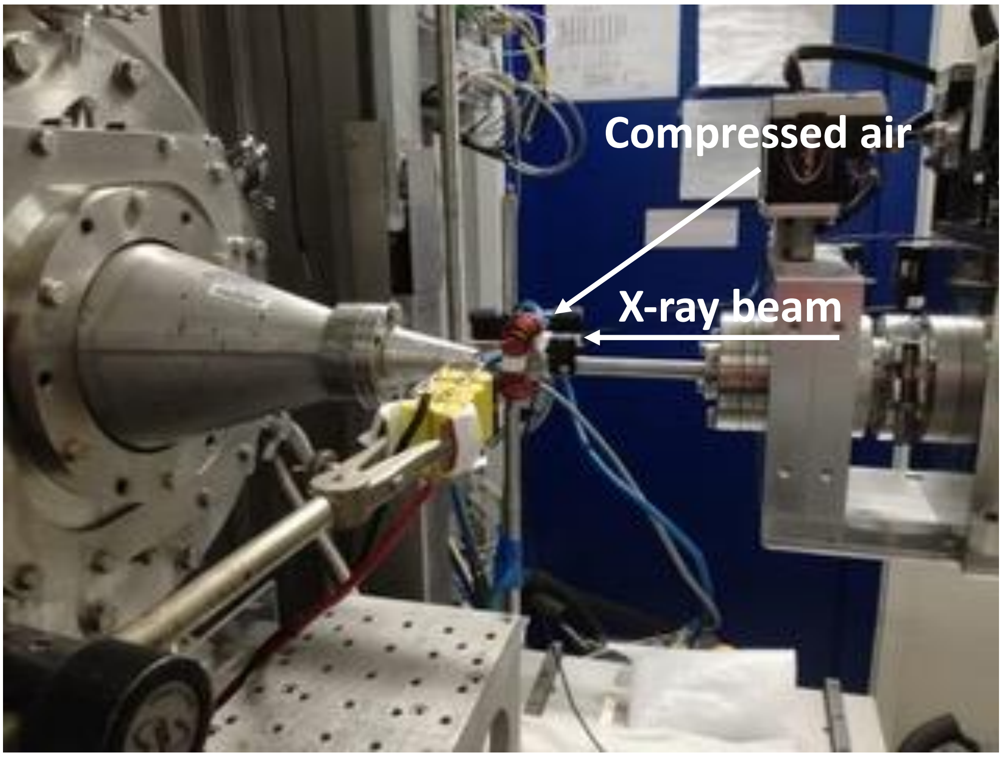

Figure S6 Experimental setup of the experiments performed on ID02 beamline ESRF Synchrotron Radiation Source. The coil was placed between the beam and the detector; the AMF was remotely controlled, and the teflon sample holder was placed in the empty space of toroidal broken part.

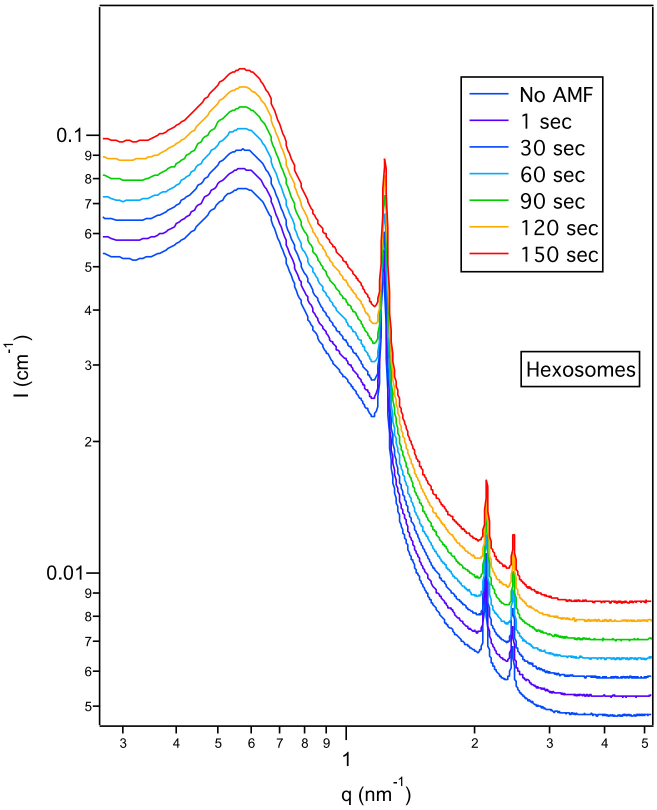

Figure S7 In situ structural detection of magnetohexosomes exposed to an oscillating magnetic field. No structural change can be seen and the lattice parameter remains constant.

Table S4 Lattice parameter (Å) of cubosomes, magnetocubosomes, magnetohexosomes exposed to an oscillating magnetic field.			
**Time (s)**	**Cubosomes**	**Magnetocubosomes**	**Magneetohexosomes**
**No AMF**	131 (Im3m)	85 (Pn3m)	51 (H_{II})
**1**	130 (Im3m)	85 (Pn3m)	51 (H_{II})
**30**	129 (Im3m)	85 (Pn3m)	51 (H_{II})
**60**	129 (Im3m)	85 (Pn3m)	51 (H_{II})
**90**	128 (Im3m)	84 (Pn3m)<br>51 (H_{II})	51 (H_{II})
**120**	128 (Im3m)	82 (Pn3m)<br>51 (H_{II})	51 (H_{II})
**150**	128 (Im3m)	51 (H_{II})	51 (H_{II})

S.8 Additional Images

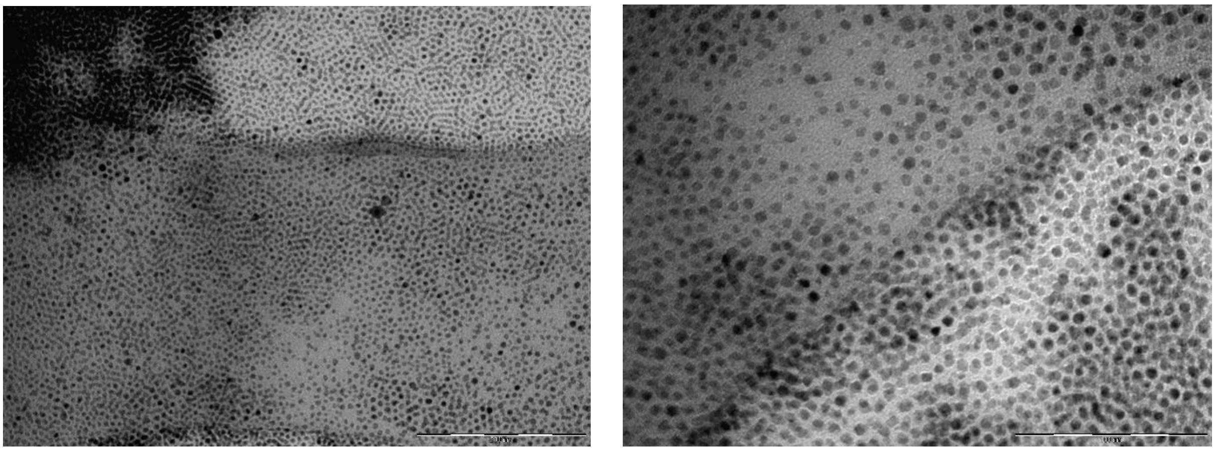

Figure S8 Representative Transmission electron microscopy (TEM) images of SPIONs at different magnifications (scale bar on the left 200 nm, scale bar on the right 100 nm). Trasmission electron microscopy (TEM) images were acquired with a STEM CM12 Philips electron microscope equipped with an OLYMPUS Megaview G2 camera, at CeME (CNR Florence Research Area, Via Madonna del Piano, 10 - 50019 Sesto Fiorentino). To the purpose drops of the SPIONs' dispersion in hexane were placed on 200 mesh carbon-coated copper grids with a diameter of 3 mm and a thickness of 50 µm (Agar Scientific) and dried at room temperature. Then, samples were analyzed at an accelerating voltage of 100 keV.

S.9 Bibliography

[1] P. Sherrer, In *Kolloidchemie Ein Lehrbuch. Chemische Technologie in Einzeldarstellungen.*, Springer, Berlin, Heidelberg, **1918**, pp. 98–100.
[2] M. Kotlarchyk, S.-H. Chen, *J. Chem. Phys.* **1983**, *79*, 2461.
[3] D. Begriff, A. Hand, D. Zerlegung, D. Fraktionierung, A. Grund, D. Fraktionierbarkeit, S. Kurven, S.- Dinger, I. Authenticated, D. Date, **1940**, *70*, 155.
